# Comparison of CD34^+^ cell enumeration between flow cytometric analysis and ADAMII-CD34 image-based fluorescence cell counter

**DOI:** 10.1371/journal.pone.0345611

**Published:** 2026-03-20

**Authors:** Anchalee Thedsawad, Sirikwan Boonmoh, Orathai Taka, Weerapat Owattanapanich

**Affiliations:** Division of Hematology, Department of Medicine, Faculty of Medicine Siriraj Hospital, Mahidol University, Bangkok Noi, Bangkok, Thailand; The Ohio State University, UNITED STATES OF AMERICA

## Abstract

**Background:**

Accurate CD34^+^ cell enumeration is essential in stem cell transplantation. Flow cytometry (FC) is the standard method to determine optimal collection time of CD34^+^ stem cells harvesting for peripheral blood stem cell (PBSC) transplantation but is costly and requires skilled personnel. The ADAMII-CD34 cell counter may be a feasible alternative for PBSC apheresis samples and for cryopreserved stem cell products which are assessed by manual cell viability counting using the trypan blue exclusion method.

**Methods:**

Fifty samples were analyzed (PBSC apheresis samples n = 25, cryopreserved stem cell products n = 25) from patients undergoing autologous PBSC transplantation or healthy donors. We assessed the quantitative agreement between methods.

**Results:**

In the 25 PBSC apheresis samples, FC-based median viable CD34^+^ absolute count was 764.7 cells/µL (IQR 453.7–1532.5; range 60.5–3233.8). ADAMII-CD34 cell counter and FC showed almost perfect agreement [Lin’s concordance correlation coefficient (CCC) 0.99 (95% CI: 0.98 to 1.00), bias –0.46% (95% CI: –5.45 to 4.53), upper limit of agreement (LoA) 22.2% (95% CI: 13.5 to 30.8), and lower LoA –23.1% (95% CI: –31.7 to –14.4)]. For cryopreserved stem cell products, viable CD34^+^ cell dose (×10^6^ cells/kg) measured by the ADAMII-CD34 cell counter and the estimated viable CD34^+^ cell dose (×10^6^ cells/kg) determined by the trypan blue exclusion method showed poor agreement [Lin’s CCC 0.58 (95% CI: 0.31 to 0.76)]. Weighted Deming regression showed proportional bias [95% CI: slope 0.02 to 0.61].

**Conclusions:**

Quantitative agreement between the ADAMII-CD34 cell counter and FC for CD34^+^ enumeration in PBSC apheresis samples was acceptable within the analyzed range. It is user-friendly and more affordable than FC. However, ADAMII-CD34 and the trypan blue exclusion method of cryopreserved stem cell products showed a proportional bias. Further considerations are needed to optimize the process for cryopreserved stem cell products.

## Introduction

Peripheral blood stem cell (PBSC) transplantation is commonly performed instead of bone marrow transplantation (BMT) for treatment of hematopoietic malignancies, and hematopoietic reconstitution after high-dose chemotherapy for solid tumors [1–4]. Collecting hematopoietic stem cells (HSCs) from peripheral blood is less invasive than from BMT, does not require anesthesia [5,6], and has an advantage in the hematopoietic function recovery rate [7,8]. Accurate determination of CD34^+^ cell numbers is clinically important and is crucial in the field of stem cell transplantation. Historically, flow cytometry has been a widely accepted method for determining the optimal harvest timing of CD34^+^ hematopoietic stem cells for both BM and PBSC transplantation [9–12]. However, this method requires expensive and complex instrumentation, involves high reagent costs, and demands specialized technical expertise. Therefore, there has been development of a tool for enumerating HSCs by measuring image based-immunofluorescence ADAMII-CD34 cell counter (NanoEntek, Seoul, Korea). This device is based on integrating computer software for digital image analysis with fluorescence microscopy. It is cost-effective, easy to use, and almost maintenance-free [13]. Furthermore, previous studies [13,14] have reported that the CD34^+^ cell counts obtained using the ADAMII-CD34 cell counter correlate strongly with those obtained by flow cytometric CD34^+^ enumeration in samples from mobilized PB and PBSC apheresis. The device requires only a small sample volume of 20 µL for analysis, significantly reducing the processing time. Results are generated and reported directly through the test-specific software, minimizing the need for user interpretation. Thus, the ADAMII-CD34 cell counter might offer accurate, convenient, and rapid enumeration of HSCs while minimizing the workload of the operator. We evaluated the quasi-quantitative agreement of CD34^+^ cell enumeration between the ADAMII-CD34 cell counter and the flow cytometric method in PBSC apheresis samples, and between the ADAMII-CD34 cell counter and manual cell viability counting using the trypan blue exclusion method in cryopreserved stem cell products.

## Materials and methods

### Blood samples

A total of 50 samples were analyzed, comprising 25 PBSC apheresis samples and 25 cryopreserved stem cell products. The PBSC apheresis samples were collected from 13 patients undergoing PBSC transplantation and 3 healthy donors, while the cryopreserved stem cell products were collected from 13 patients and 4 healthy donors. Twelve patients were represented in both sample groups. Both groups included 4 patients with multiple myeloma (MM) and 9 patients with lymphoma. These samples were obtained from residual specimens of autologous HSC transplantation in patients with hematologic malignancy, allogeneic donors for routine CD34^+^ enumeration by flow cytometry, and the trypan blue exclusion method at the Division of Hematology, Department of Medicine, Faculty of Medicine Siriraj Hospital, Mahidol University, Bangkok, Thailand. This study was conducted in accordance with the Declaration of Helsinki (as revised in 2013). The protocol was approved by the Siriraj Institutional Review Board (SIRB), Faculty of Medicine Siriraj Hospital, Mahidol University, Bangkok, Thailand (reference: Si 499/2023). The IRB approved a waiver of written informed consent due to the use of anonymized leftover samples collected after routine clinical laboratory analysis. No identifiable personal data were included. Blood samples were accessed for research purposes from 01/08/2023–30/11/2023.

Our institutional clinical thresholds define PBSC mobilization adequacy as a pre-apheresis peripheral blood CD34^+^ cell count ≥ 20 cells/µL by flow cytometry, with a minimum target transplant dose of ≥ 2 × 10^6^ CD34^+^ cells/kg of the recipient’s body weight. Additional apheresis sessions were performed when the collected CD34^+^ cell dose was below this minimum target.

### CD34^+^ cell enumeration by flow cytometry in the PBSC apheresis group

Leukapheresis products from the PBSC apheresis group were diluted 1:10 with 1 × phosphate-buffered saline (PBS), and counted using an automated blood cell counter. After dilution, the white blood cell (WBC) count was required to be less than 40,000 cells/μL. BD stem cell enumeration (SCE) kit (BD Biosciences, San Jose, CA) reagents were used in this study. Daily quality control using Cytometer Setup and Tracking (CS&T) beads (BD Biosciences, San Jose, CA) was performed prior to sample analysis to ensure acceptable instrument performance. For CD45/CD34 surface antigen staining, a lyse-no-wash protocol was used. Viable CD34^+^ cells were defined as 7-AAD–negative events within the CD34^+^ gate, following standard ISHAGE gating. Absolute CD34^+^ counts were calculated using BD Trucount beads (BD Biosciences). Briefly, 20 μL of stem cell reagent and 20 μL of 7-AAD were added to a BD Trucount tube containing 100 μL of a 1:10 diluted sample from the PBSC apheresis product. The mixture was incubated at room temperature in the dark for 20 minutes. To enhance the accuracy and reproducibility of measurements, samples were added to antibody mix using reverse pipetting. Following incubation, 2 mL of ammonium chloride was added to lyse red blood cells. The tubes were gently vortexed and incubated in the dark for 10 minutes. Samples were analyzed within one hour using a BD FACSVerse flow cytometer (BD Biosciences). The computed results were represented as viable CD34^+^ absolute count (cells/µL), viable CD45^+^ absolute count (cells/µL), and percentage of viable CD34^+^ within the viable CD45^+^ population.

### Manual cell viability counting using the trypan blue exclusion method in the cryopreserved stem cell product group

Cell viability was assessed by the trypan blue exclusion method and a hemocytometer. Mononuclear cells (MNCs) were first isolated from the cryopreserved stem cell products prior to staining. This step ensures that the measured percentage of viable cells reflects primarily the MNC population and minimizes interference from granulocytes, which are more susceptible to cryopreservation-induced cell damage. To a 50 mL centrifuge tube, 7.5 mL of Ficoll-Hypaque separation (IsoPrep, Robbins Scientific Corporation, Sunnyvale, CA) was added. In a separate 15 mL tube, 0.5 mL of cryopreserved stem cell products was mixed with 1 × PBS to a total volume of 15 mL. The mixture was carefully layered onto Ficoll-Paque and centrifuged at 2000 rpm for 20 minutes at 4°C. The mononuclear cell layer was aspirated, transferred to a new tube, washed with 10 mL of 1 × PBS, and centrifuged for 5 minutes. Next, the supernatant was discarded, and the pellet was resuspended in 0.3 mL of 1 × PBS. From the resuspended cell sample, 10 μL was stained with 40 μL of 0.4% Trypan Blue (TB) solution (Gibco, Life Technologies Corporation, Waltham, MA). Next, 10 μL of the mixture was pipetted into a hemocytometer chamber, and cells were counted using a brightfield microscope at 40 × magnification. Within 2–5 minutes, cells were counted in each of the four outer squares of the hemocytometer. The total number of cells was recorded; blue-stained cells were counted as dead (non-viable), whereas unstained (transparent) cells were counted as live (viable). The percentage of cell viability was calculated using the following formula: (number of viable cells/ total number of cells) × 100. The estimated viable CD34^+^ cell dose per kilogram of recipient’s body weight was calculated by multiplying the pre-freeze viable CD34^+^ cell dose (×10^6^ cells/kg), measured by flow cytometry, by the percentage of viable MNCs post-thaw (including CD34^+^ cells, lymphocytes, and monocytes) determined by trypan blue exclusion.

### CD34^+^ cell enumeration by the ADAMII-CD34 cell counter in the PBSC apheresis and cryopreserved stem cell product groups

Before sample staining, leukapheresis or cryopreserved stem cell products were diluted with 1 × PBS to adjust the total white blood cell (WBC) count of the sample to 150,000 cells/μL. All experiments were performed within 24 hours of collection. The ADAMII-CD34 cell counter is a four-channel, image-based fluorescence cell counter designed for bench-top use. It detects bright-field (cells/debris, size, and shape), green (fluorescein isothiocyanate [FITC]-live/dead marker with SYTOX blue), yellow (phycoerythrin [PE]-conjugated anti-CD34 antibody), and red (peridinin chlorophyll protein complex [PerCP]-conjugated anti-CD45 antibody). The ADAMII-CD34 instrument was calibrated using ADAMII Calibration beads (NanoEntek, Korea) prior to the first use, and quality control was performed using CD-Chex CD34 Flow Cytometry Controls (Streck, NE) before sample analysis to ensure accurate and consistent instrument performance. Briefly, 20 μL diluted sample was incubated for 20 minutes in the dark at room temperature with 5 μL of reagent containing fluorescence-labeled antibodies and SYTOX blue nucleic acid dye from the ADAMII-CD34 reagent kit (NanoEntek, Seoul, Korea). After staining, red blood cells (RBCs) were lysed with 1 × RBC lysis buffer for 10 minutes under the same conditions. The 25-μL lysed sample was loaded into an ADAMII assay slide (NanoEntek) and placed on the slide holder of the ADAMII instrument. The ADAMII captures images through each fluorescence channel and analyzes them to enumerate total and fluorescence-labeled cells based on size, shape, viability, and surface marker. Measured parameters in cells/μL include viable CD34^+^ cell count, viable CD45^+^ cell count, total CD34^+^ cell count, and total CD45^+^ cell count, along with CD34^+^ cell viability (%), CD45^+^ cell viability (%), and the percentage of viable CD34^+^ cells of viable CD45^+^ cells (CD34^+^/CD45^+^, %).

In the cryopreserved stem cell product group, the percentage of viable MNCs post-thaw determined by the trypan blue exclusion method and the viable CD34^+^ cell count (cells/µL) measured by the ADAMII-CD34 cell counter were converted to the estimated viable CD34^+^ cell dose and the viable CD34^+^ cell dose (×10^6^ cells/kg of recipient’s body weight), respectively, for comparison.

### Statistical analysis

We performed quantitative comparisons of agreement. Parametric distribution was assessed by graphical analysis and the Shapiro-Wilk test [15]. Descriptive statistics are presented as median (Q1, Q3) and range. Correlation analysis was performed to assess linear association. A correlation coefficient (*r*) of 0.90–1.00 is a very strong correlation; *r* of 0.70–0.89 is a strong correlation; *r* of 0.40–0.69 is a moderate correlation; *r* of 0.10–0.39 is a weak correlation; and *r* of 0.00–0.09 is a negligible correlation. Enumeration methods were compared using Wilcoxon’s signed-rank test for paired data. Quantitative agreement analysis was assessed by Lin’s concordance correlation coefficient (Lin’s CCC) [16]. For interpretation of Lin’s CCC, > 0.99 is almost perfect agreement; 0.95–0.99 is substantial agreement; 0.90–0.95 is moderate agreement; and < 0.90 is poor agreement [17]. Evaluation for systematic and proportional bias were assessed by unweighted and weighted Deming regression with jackknifed standard errors [18]. Deming regression results were interpreted as showing no systematic bias if the 95% confidence interval (CI) of the intercept included zero, and no proportional bias if the 95% CI of the slope coefficient included one. Difference plots showing Bland-Altman horizontal bias and limits of agreement of percentage difference results were obtained if assumptions were met. Proportional bias was tested, and if the test was significant, we used linear regression to plot the proportional bias [19]. The main analysis retained all samples. The potential influence of outliers was assessed in exploratory analyses [19], and outliers for these analyzes were identified using Grubbs’ test and graphical assessment of percentage-difference values [20].

Statistical analysis was performed using R 4.4.2 (R Core Team, 2025, R Foundation for Statistical Computing, Vienna, Austria), with the packages *mcr* (version 1.3.3.1), *nortest* (version 1.0–4), *ggplot2* (version 3.5.1), *outliers* (version 0.1.5), and *DescTools* (version 0.99.60); and Stata 15.1 (StataCorp LLC., 2017, College Station, TX, USA) with the user-written command *blandaltman*. A *p*-value <0.05 was considered significant.

## Results

The 25 PBSC apheresis samples and 25 cryopreserved stem cell products, obtained from 4 healthy donors, 5 samples from patients with MM, and 16 samples from patients with lymphoma, were used for two methods of quantitative agreement analysis. The median age was 52 years (range 13 − 65), with a female predominance in both groups.

### Correlation and agreement between flow cytometry and ADAMII-CD34 cell counter in the PBSC apheresis group

**Fig 1:**
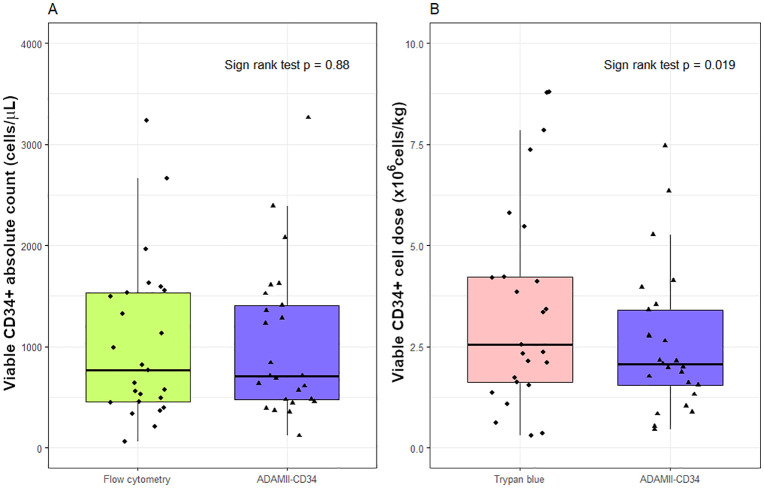
Boxplots of distributions of CD34^+^ enumerations by measurement device of (A) PBSC apheresis and (B) cryopreserved stem cell products: ADAMII-CD34 viable CD34^+^ cell dose and Trypan blue estimated viable CD34^+^ cell dose (×10^6^ cells/kg).

In all 25 samples, there were no significant differences between the ADAMII-CD34 cell counter and flow cytometry in median viable CD34 ⁺ absolute counts [709.6 cells/µL (IQR 477.4–1408.3; range 120.7–3264.7) vs. 764.7 cells/µL (IQR 453.7–1532.5; range 60.5–3233.8); *p* = 0.882] (Fig 1A), median viable CD45 ⁺ absolute counts [210,937.6 cells/µL (IQR 175,026.3–255,497.1; range 115,181.4–509,060.2) vs. 215,969.8 cells/µL (IQR 166,985.2–267,912.1; range 110,354.2–463,298.4); *p* = 0.925], and the percentage of viable CD34 ⁺ cells within the viable CD45 ⁺ population [0.33% (IQR 0.27–0.67; range 0.05–1.00) vs. 0.40% (IQR 0.26–0.61; range 0.03–1.10); *p* = 0.721] as shown in Table 1.

**Table 1:** Comparison of viable CD45^+^ absolute count, viable CD34^+^ absolute count (cells/µL), %viable CD34^+^ within viable CD45^+^ between the ADAMII-CD34 cell counter and flow cytometry in PBSC apheresis, and viable CD34^+^ cell dose and estimated viable CD34^+^ cell dose (×10^6^ cells/kg) between the ADAMII-CD34 cell counter and the trypan blue exclusion method in cryopreserved stem cell product groups.

Parameter	PBSC apheresis						Cryopreserved stem cell products		
	All samples			Samples ≤ 1000 cells/µL			All samples		
	ADAMII-CD34 (n = 25)	Flow cytometry (n = 25)	*p*-value	ADAMII-CD34 (n = 15)	Flow cytometry (n = 15)	*p*-value	ADAMII-CD34 (n = 25)	Trypan blue (n = 25)	*p*-value
**Viable CD45** ^**+**^ **absolute count (cells/µL),** **median (IQR) [range]**	210,937.6 (175,026.3, 255,497.1) [115,181.4, 509,060.2]	215,969.8 (166,985.2, 267,912.1) [110,354.2, 463,298.4]	0.925	202,776.8 (139,534.9, 227,660.1) [110,354.2, 360,534.1]	194,555.6 (161,554.3, 236,647.1) [110,354.2, 115,181.4]	0.307			
**Viable CD34** ^**+**^ **absolute count (cells/µL),** **median (IQR) [range]**	709.6 (477.4, 1408.3) [120.7, 3264.7]	764.7 (453.7, 1532.5) [60.5, 3233.8]	0.882	479.2 (391.3, 689.5) [120.7, 841.3]	494.2 (369.3, 642.3) [60.5, 989.3]	0.427			
**%Viable CD34**^**+**^ **within viable CD45**^**+**^ **median (IQR) [range]**	0.33 (0.27, 0.67) [0.05, 1.00]	0.40 (0.26, 0.61) [0.03, 1.10]	0.721	0.30 (0.19, 0.33) [0.05, 0.48]	0.28 (0.20, 0.39) [0.03, 0.55]	0.975			
**Viable CD34** ^**+**^ **cell dose** **(×10**^**6**^ **cells/kg)**^**b**^, **median (IQR) [range]**							2.07 (1.55, 3.41) [0.45, 7.46]		0.019^a^
**Estimated viable CD34** ^**+**^ **cell dose (×10**^**6**^ **cells/kg)**^**c**^, **median (IQR) [range]**								2.56 (1.62, 4.23) [0.31, 8.80]	

*P*-values are Wilcoxon Signed Rank test. ^a^Significant at the < 0.05 level. ^b^Viable CD34^+^ cell dose (×10^6^ cells/kg) from the ADAMII-CD34 cell counter was calculated based on viable CD34^+^ absolute counts. ^c^Estimated viable CD34^+^ cell dose (×10^6^ cells/kg) the trypan blue exclusion method was derived from %MNC viability.

**Fig 2:**
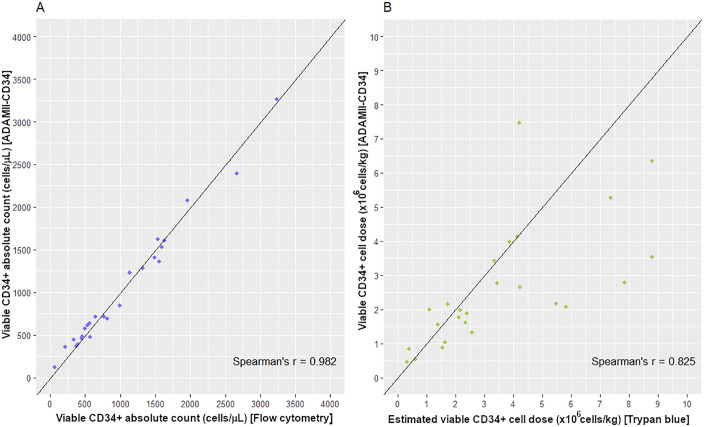
Scatterplots of CD34^+^ enumerations of (A) PBSC apheresis and (B) cryopreserved stem cell products.

Correlation between flow cytometry and ADAMII-CD34 cell counter was very strong (*r*_*s*_ = 0.98), and the scatterplot suggested neither systematic nor proportional bias (Fig 2A).

**Fig 3:**
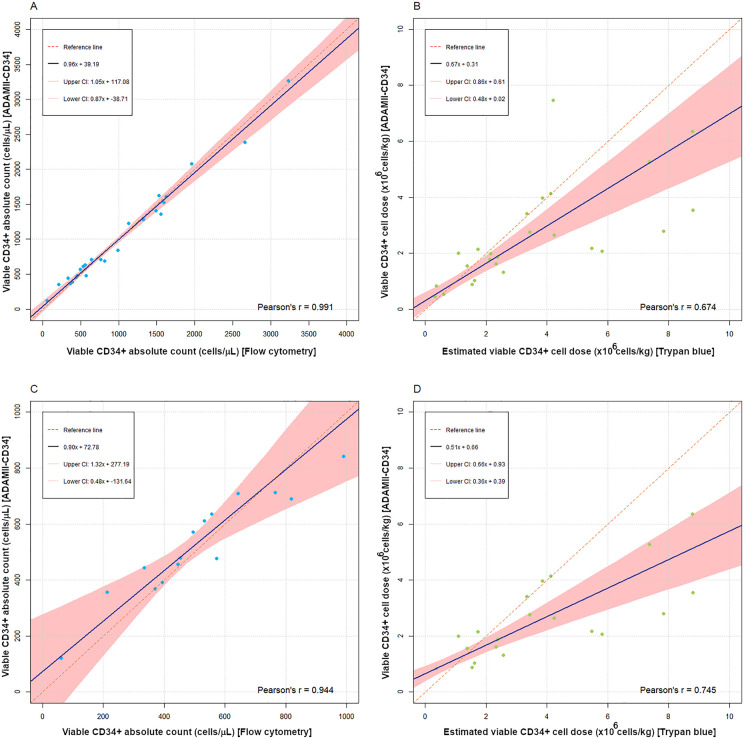
Deming regression for systematic and proportional bias assessment. **(A)** Unweighted Deming regression for PBSC apheresis. **(B)** Weighted Deming regression for cryopreserved stem cell products. **(C)** Unweighted Deming regression for PBSC apheresis samples ≤ 1000 cells/µL. **(D)** Weighted Deming regression of cryopreserved stem cell products without outliers. **Abbreviation:** CI, confidence interval.

**Fig 4:**
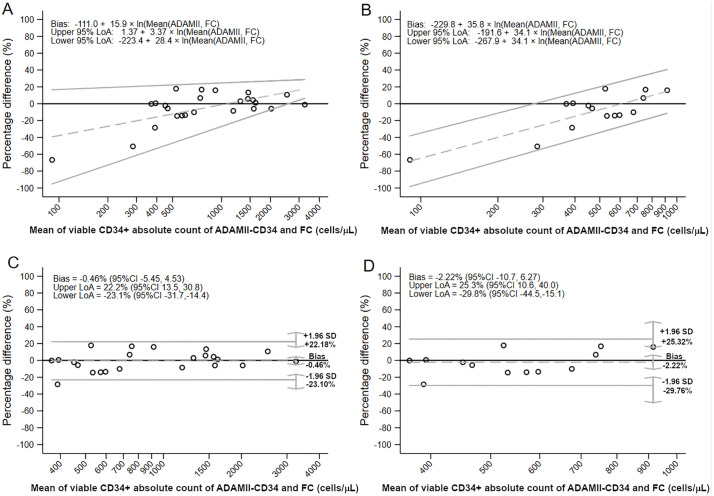
Difference plots showing the percentage difference between flow cytometry and the ADAMII-CD34 cell counter for (A) PBSC apheresis with regression-based proportional bias and limits of agreement in the total range, (B) PBSC apheresis with regression-based proportional bias and limits of agreement in mean CD34^+^ count ≤ 1000 cells/µL, (C) PBSC apheresis with Bland-Altman limits of agreement with two outliers excluded in the total range, and (D) PBSC apheresis with Bland-Altman limits of agreement with two outliers excluded ≤ 1000 cells/µL. Percentage difference (%) was calculated as: [(Flow cytometry – ADAMII-CD34)/ ((Flow cytometry + ADAMII-CD34)/ 2)] × 100. Dashed grey lines are mean bias. Solid grey lines are upper and lower limits of agreement. The axes of the means are on a log scale. **Abbreviations:** CI, confidence interval; FC, flow cytometry; LoA, limit of agreement; SD, standard deviation.

In quantitative agreement analysis, Lin’s CCC showed almost perfect agreement [Lin’s CCC 0.99 (95% CI: 0.98 to 1.00)]. The linearity assumption assessment for unweighted Deming regression was acceptable (*r*_*p*_ = 0.99) (Fig 3A). The 95% CI for both the intercept and slope of the unweighted Deming regression included the null values, indicating no evidence of systematic or proportional bias (intercept 95% CI: –38.7 to 117.1; slope 95% CI: 0.87 to 1.05) (Fig 3A). Normality of residuals and homogeneity of variance checks for Deming regression in S1A and S1B Fig demonstrate these assumptions were met. Because absolute difference was transformed to percentage difference, we performed Bland-Altman analysis assumption checks and outlier identification. There was a significant nonparametric distribution of percentage difference (*p* = 0.001) and a significant proportional bias (*p* = 0.049), with the ADAMII-CD34 cell counter tending to count higher values than flow cytometry in the low measurement range (mean of the two methods < 600 cells/μL) (Fig 4A and 4B). Grubbs’ test identified significant outliers (*p* < 0.001), indicating the two lowest mean viable CD34^+^ absolute counts were outliers, which were excluded to explore their influence. Retesting of proportional bias was nonsignificant (*p* = 0.29) and the percentage difference showed a parametric distribution (*p* = 0.74). The Bland-Altman mean bias was –0.46% (95% CI: –5.45 to 4.53) (Fig 4C). The upper limit of agreement was 22.2% (95% CI: 13.5 to 30.8), and the lower limit of agreement was –23.1% (95% CI: –31.7 to –14.4) (Fig 4C).

We performed a subgroup analysis of samples with viable CD34^+^ absolute counts of ≤ 1000 cells/µL, which is the lower range category in the present study. In 15 samples, the median viable CD34^+^ absolute counts were not significantly different between the ADAMII-CD34 counter and flow cytometry [479.2 cells/μL (IQR 391.3–689.5; range 120.7–841.3) vs. 494.2 cells/μL (IQR 369.3–642.3; range 60.5 − 989.3), respectively; *p* = 0.43]. Median viable CD45^+^ absolute counts and the percentage of viable CD34^+^ within viable CD45^+^ were also not significantly different (Table 1). Correlation between flow cytometry and the ADAMII-CD34 cell counter was very strong (*r*_*s*_ = 0.94). Lin’s CCC showed moderate agreement [Lin’s CCC 0.91 (95% CI: 0.80 to 0.96)]. The linearity assumption assessment for unweighted Deming regression was acceptable (*r*_*p*_ = 0.94) (Fig 3C). The 95% CI for both the intercept and slope of the unweighted Deming regression did not show systematic or proportional bias (intercept 95% CI: –131.6 to 277.2; slope 95% CI: 0.48 to 1.32) (Fig 3C). Normality of residuals and homogeneity of variance assumptions for Deming regression were acceptable (S2A and S2B Fig). For Bland-Altman analysis, the two aforementioned outliers were excluded. Thus, 13 samples were analyzed. A Linear regression test to check for proportional bias in percentage difference before reporting horizontal Bland-Altman limits of agreement was nonsignificant (*p* = 0.081), and the percentage difference was parametrically distributed (*p* = 0.56). The Bland-Altman parametric mean bias –2.22% (95% CI: –10.7 to 6.27) (Fig 4C). The upper limit of agreement was 25.3% (95% CI: 10.6 to 40.0), and the lower limit of agreement was –29.8% (95% CI: –44.5 to –15.1) (Fig 4D).

### Correlation and agreement between manual cell viability counting using the trypan blue exclusion method and ADAMII-CD34 cell counter in cryopreserved stem cell product groups

The median viable CD34^+^ cell dose derived from the ADAMII-CD34 cell counter was significantly different from that estimated by the trypan blue exclusion method [2.07 × 10^6^ cells/kg (IQR 1.55–3.41; range 0.45–7.46) vs. 2.56 × 10^6^ cells/kg (IQR 1.62–4.23; range 0.31–8.80), respectively; *p* = 0.019] (Table 1 and Fig 1B).

The correlation between the trypan blue exclusion method and the ADAMII-CD34 cell counter was moderate (*r*_*s*_ = 0.83). The scatterplot suggested proportional bias and heteroscedasticity with increasing magnitude of values (Fig 2B).

In quantitative agreement analysis, Lin’s CCC showed poor agreement [Lin’s CCC 0.58 (95% CI: 0.31 to 0.76)]. The linearity assumption assessment for weighted Deming regression was acceptable (*r*_*p*_ = 0.67) (Fig 3B). The 95% CI of both the intercept and slope did not include the null values, indicating both systematic and proportional bias (intercept 95% CI: 0.02 to 0.61; slope 95% CI: 0.48 to 0.86) (Fig 3B), with the trypan blue exclusion method tending towards higher counts than the ADAMII-CD34 cell counter. Normality of residuals and homogeneity of variance checks for Deming regression in S3A and S3B Fig showed that the assumption of normality of residual error was not met (*p* = 0.004). Thus, we performed a sensitivity analysis excluding outliers identified by Tukey’s method in S4 Fig. After re-checking the assumptions of the Deming model (S5A and S5B Fig), we re-performed weighted Deming regression, which revealed results similar to those of the model that included outliers (Fig 3D). We did not report difference plots with horizontal or non-horizontal bias and limits of agreement due to a lack of clinical interpretability.

## Discussion

The present study confirms a very strong correlation in the measurement of HSCs using flow cytometry and ADAMII-CD34 cell counter in PBSC apheresis samples. This finding is consistent with previous studies by Rah et al. [13] and Yu et al. [14], both of which demonstrated that ADAMII had excellent correlation for CD34^+^ cell enumeration in leukapheresis samples. The two methods for enumerating CD34^+^ cells are based on staining with labeled monoclonal antibodies against CD34 and CD45, but differ in their analytic approaches. Flow cytometry measures individual cells as they flow through the flow cell, using forward scatter, side scatter, fluorescently labeled antibodies against CD34/CD45, and viability dye staining. The sheath fluid generates a controlled stream of cells or particles. Enumeration of CD34^+^ cells by flow cytometry also relies on synthetic fluorescent microspheres as internal counting controls, with cells and counting beads analyzed simultaneously. Gating strategies are used to identify viable CD34^+^ cells. Conversely, the ADAMII-CD34 cell counter uses a fluorescence microscope with Light Emitting Diode excitation and Charge-Coupled Device detection technologies to capture and analyze images to enumerate the total and fluorescence-labeled cells based on size, shape, viability, and surface markers. It is easy to use, cost-effective, and suitable for less experienced laboratory personnel due to its built-in optics and analysis software. It is also less complicated than flow cytometry in both usage and analysis, as it does not require compensation or gating analysis. However, autofocusing must be performed before measurement by ADAMII-CD34 cell counter to ensure clear images. If the images are unclear, it can affect the accuracy of CD34^+^ cell enumeration and may lead to an overcount due to debris that might also be included in the count.

To date, there is no evidence-based widespread consensus on the acceptable percentage difference in quasi-quantitative comparison of CD34^+^ counting methods. Nevertheless, it has recently been suggested that interlaboratory comparative performance in flow cytometry could be evaluated using allowable total error (aTE) at the 80^th^ percentile derived from state-of-the-art (SOTA) total error analysis of external quality assurance schemes (EQAS) [21]. A recent Spanish EQAS data study of flow cytometry methods across 40 laboratories, using unstabilized PBSC samples sent to them enumerated within 24 hours of arrival, reported a SOTA-based acceptable total error (%aTE) at the 80^th^ percentile of 21.1% for samples with enumerations of absolute CD34^+^ counts > 25 cells/µL (range: 25.03–132.9 cells/µL). The study used data from participating laboratories collected between 2017–2020, which reflects recent practice standards in flow cytometry. The investigators also reported that %aTE at the 80^th^ percentile decreased as absolute CD34^+^ cell counts increased [22]. Our comparison is similar in that we used unstabilized samples enumerated within 24 hours of collection and removed outliers in the statistical analysis, using a contemporary flow cytometry method. However, our study differs in terms of the range of absolute CD34^+^ cell counts investigated. In analysis of all PBSC apheresis samples, we found significant proportional bias in the low measurement range (mean of the two methods < 600 cells/µL). We also performed an alternative analysis excluding outliers to explore their influence and found that their removal allowed for meeting the assumptions of Bland-Altman analysis to estimate mean difference and limits of agreement. We found that both the bias and the upper and lower LoAs across the full analytical range were acceptable, based on a %aTE at the 80^th^ percentile of 21.1%, with point estimates of the upper and lower LoAs of 22.2% and −23.1%, respectively. Results for the percentage difference were quite similar in our range-restricted analysis (≤ 1000 cells/µL). It is not generally recommended to exclude outliers from Bland-Altman analysis, but it has been suggested that their influence may be explored [19]. To the best of our knowledge, Yu et al. [14] has presented Bland-Altman analysis of the ADAMII-CD34 cell counter compared to flow cytometry in the literature. They used flow cytometry values as a gold standard reference measurement method with many flow cytometry count values in the range < 300 cells/µL. Within this range, increased variability both in positive and negative percentage differences were found compared to count values ≥ 300 cells/µL, demonstrating that ADAMII-CD34 cell counter gave both higher and lower counts than flow cytometry. Thus, we suggest that our study is limited by the small number of PBSC apheresis samples in this lower range, and the proportional bias that we detected may be due to this. These outlier-excluded results should be interpreted with caution due to their removal. Future studies should collect a large sample size of PBSC apheresis samples across the total range, and especially in the lower range to confirm these results.

The cause of the outliers in the present study is presumed to be the low number of CD34^+^ cells in the samples. The ADAMII-CD34 cell counter detects cells using software-based analysis of static fluorescence images, which may misclassify non-specific antibody binding or autofluorescence as CD34^+^ cells. In contrast, flow cytometry analyzes each cell as it passes through a laser beam and allows for gating and compensation, which helps reduce interference from non-specific signals. This may have resulted in higher viable CD34^+^ absolute counts analyzed by the ADAMII-CD34 cell counter (Outliers 1 and 2: 120.71 and 356.64 cells/μL, respectively) compared to flow cytometry (60.45 and 212.74 cells/μL, respectively), particularly in samples with low CD34^+^ cell counts. However, further studies with a larger sample size in this population may be required to support this assumption more conclusively.

We observed significant differences in viable CD34^+^ cell dose (×10^6^ cells/kg) between the estimated viable CD34^+^ cell dose derived from the trypan blue exclusion method and the viable CD34^+^ cell dose measured by the ADAMII-CD34 cell counter method in samples collected from cryopreserved stem cell products. Our results showed poor agreement by Lin’s CCC between the two methods. In terms of acceptability, the slope also deviates from zero, suggesting that the trypan blue exclusion method tends towards higher counts compared to the ADAMII-CD34 cell counter. Furthermore, a systematic bias is suggested by the weighted Deming regression. Nevertheless, these interpretations should be made with caution due to the relatively small sample size of this analysis, and further studies are required. The observed biases between the two methods may have resulted from differences in the values used to calculate viable CD34^+^ cell counts. The trypan blue exclusion method estimates viable CD34^+^ cells based on mononuclear cell (MNC) counts, which primarily include CD34^+^ cells, but there may also have been lymphocytes and monocytes present. Conversely, the ADAMII-CD34 cell counter directly measures viable CD34^+^ cell counts. Therefore, while the ADAMII-CD34 cell counter provides a direct count, the trypan blue exclusion method derives viable CD34^+^ cell counts from MNC viability percentages. In cryopreserved stem cell products, viability assessment focused on the MNC fraction to minimize interference from cryo-sensitive granulocytes. The estimated viable CD34^+^ cell dose was calculated using post-thaw MNC viability by the trypan blue exclusion method. This approach provides a more accurate reflection of CD34^+^ progenitor survival than total nucleated cell (TNC) viability, as granulocytes and other non-MNCs are highly sensitive to cryopreservation-induced damage and may underestimate CD34^+^ cell survival. The trypan blue exclusion method assumes proportional survival of CD34^+^ cells within the MNC population and evaluates overall membrane integrity; therefore, this estimation should be interpreted as an approximate reflection of CD34^+^ progenitor survival rather than a direct measurement of CD34^+^ viability. This is consistent with clinical practice guidelines [23], which prioritize pre-cryopreservation TNC dose and HLA matching as the primary criteria for graft selection rather than post-thaw TNC viability. Post-thaw TNC viability may not reliably predict clinical engraftment. By focusing on the MNC fraction, our approach partially mitigates the limitations inherent in total cell-based assessments, which are often confounded by cryo-sensitive non-progenitor cells.

Providing biological context for this approach, Cai et al. [24] demonstrated that T cells and granulocytes from PBSC or PBMC apheresis products are more susceptible to freeze–thaw-induced damage, resulting in reduced post-thaw viability. In contrast, other cell populations, including hematopoietic stem/progenitor cells, B cells, NK cells, and monocytes, appear to be more resistant to cryopreservation, maintaining higher viability after thawing. Likewise, Vrhovac et al. [25] observed that hematopoietic progenitor cells (HPCs) exhibit greater resistance to cryopreservation-induced damage, resulting in higher post-thaw viability compared with total nucleated cells. These observations support the assumption that CD34^+^ cells may retain relatively greater viability within the MNC population following cryopreservation.

Although the detection of viable CD34^+^ cell in cryopreserved stem cell products has not yet been standardized, flow cytometry is the most commonly used technique for CD34^+^ enumeration in cryopreserved stem cell products [26–28], with trypan blue occasionally used for viability testing [29]. Despite its widespread use, flow cytometry has limitations for cryopreserved stem cell products due to dimethyl sulfoxide (DMSO), which protects cells during freezing by preventing ice crystal formation. However, upon thawing, DMSO becomes cytotoxic, accelerating cell degradation, apoptosis, and oxidative stress, which can damage DNA and proteins. Minimizing residual DMSO before testing may be essential, and can be achieved by washing cells with solutions like MEM-BSA, 1% BSA-PBS [30], RPMI, or DMEM to reduce cytotoxicity, maintain osmotic balance, and enhance viability. However, most clinics do not calculate the CD34^+^ count before frozen products are thawed for HSCT and before they are administered to the patient [27]. Therefore, only a few studies have compared the DMSO removal process [27,31].

Meriç et al. [27] reported that viable CD34^+^ stem cell counts increased significantly after DMSO removal compared to unwashed samples. These findings suggest that DMSO removal improves the accuracy of viable CD34^+^ cell analysis. However, to enhance CD34^+^ cell detection in cryopreserved stem cell products, future protocols should include a DMSO washing step prior to analysis using the ADAMII-CD34 cell counter to reduce cell damage and improve accuracy.

Another possible reason for the proportional bias observed between the ADAMII-CD34 cell counter and the trypan blue exclusion method for cryopreserved stem cell products is the post-thaw processing time, which should be minimized to ensure accurate results. In this study, viable CD34^+^ cell counts obtained using the trypan blue exclusion method were higher than those obtained using the ADAMII-CD34 cell counter, possibly due to differences in testing duration, as the trypan blue exclusion method takes less than five minutes, whereas the ADAMII-CD34 cell counter requires approximately 40 minutes. During the antibody incubation step in the ADAMII-CD34 cell counter, residual DMSO may have had an adverse effect on the cells, leading to a lower viable CD34^+^ cell count.

In conclusion, this study confirms the ADAMII-CD34 cell counter shows almost perfect agreement with flow cytometry for PBSC apheresis samples, Proportional bias with ADAMII-CD34 cell counter tending to give higher counts in the low measurement range was detected, although this may have been due to influential outliers. Bland-Altman analysis showed acceptable mean differences and limits of agreement in exploration of their influence by excluding them. The method is user-friendly, both in terms of the analysis process and result interpretation. In addition, the equipment is more affordable compared to flow cytometry. While the ADAMII-CD34 cell counter may not be suitable for cryopreserved stem cell products, implementing a DMSO removal step in the pre-analytic phase and minimizing the time to analysis may improve accuracy.

## Supporting information

S1 Fig Model assumption checks for the unweighted Deming regression model of flow cytometry vs. the ADAMII-CD34 cell counter for PBSC apheresis samples. (A) Normality of residuals. (B) Homogeneity of variance.
(TIF)

S2 Fig Model assumption checks for the unweighted Deming regression model of flow cytometry vs. the ADAMII-CD34 cell counter for PBSC apheresis samples ≤ 1000 cells/µL. (A) Normality of residuals. (B) Homogeneity of variance.
(TIF)

S3 Fig Model assumption checks for the weighted Deming regression model of trypan blue exclusion method vs. ADAMII-CD34 cell counter including outliers for cryopreserved stem cell products. (A) Normality of residuals. (B) Homogeneity of variance.
(TIF)

S4 Fig Boxplot of residuals for weighted Deming regression of cryopreserved stem cell products to identify outliers. (TIF)

S5 Fig Model assumption checks for the weighted Deming regression model of trypan blue exclusion method vs. ADAMII-CD34 cell counter without outliers for cryopreserved stem cell products. (A) Normality of residuals. (B) Homogeneity of variance.
(TIF)

S6 Dataset Minimal dataset for this study, containing laboratory parameters for PBSC apheresis samples and cryopreserved stem cell products in separate sheets. (XLS)
