# Supplementary figures and images for: Comparison of CD34+ cell enumeration between flow cytometric analysis and ADAMII-CD34 image-based fluorescence cell counter
Source: PLoS One. 2026 Mar 20;21(3):e0345611. doi: 10.1371/journal.pone.0345611 (PMC13004359; doi:10.1371/journal.pone.0345611)

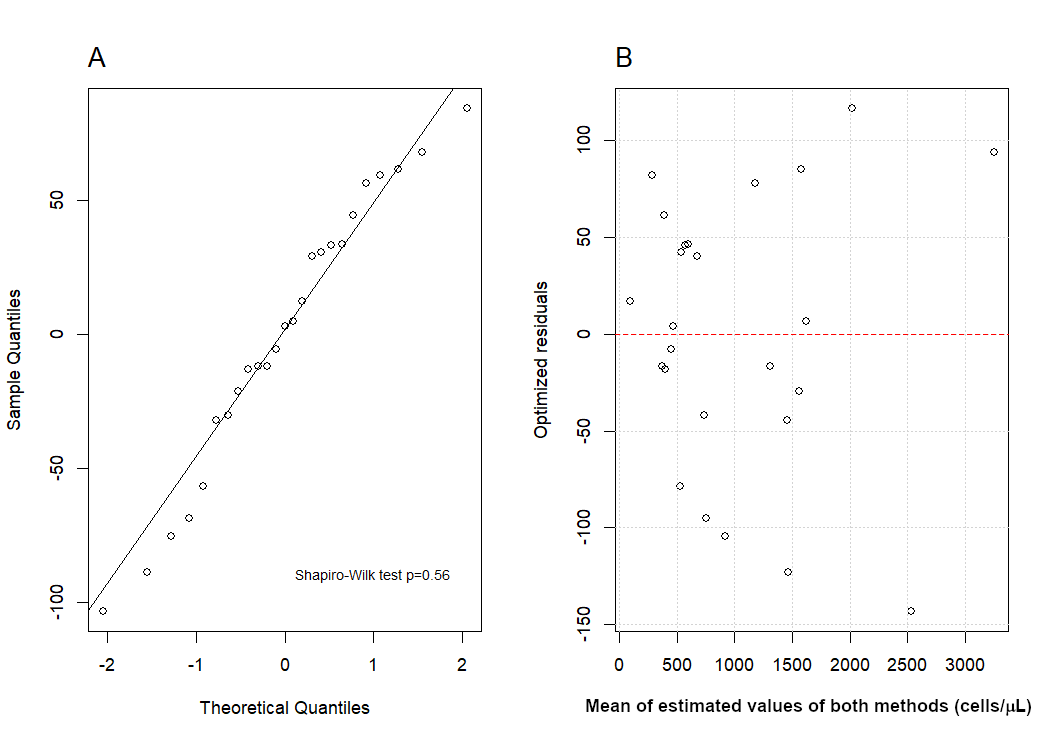

Supplement: S1 Fig — (A) Normality of residuals. (B) Homogeneity of variance. (TIF) [file pone.0345611.s001.tif]

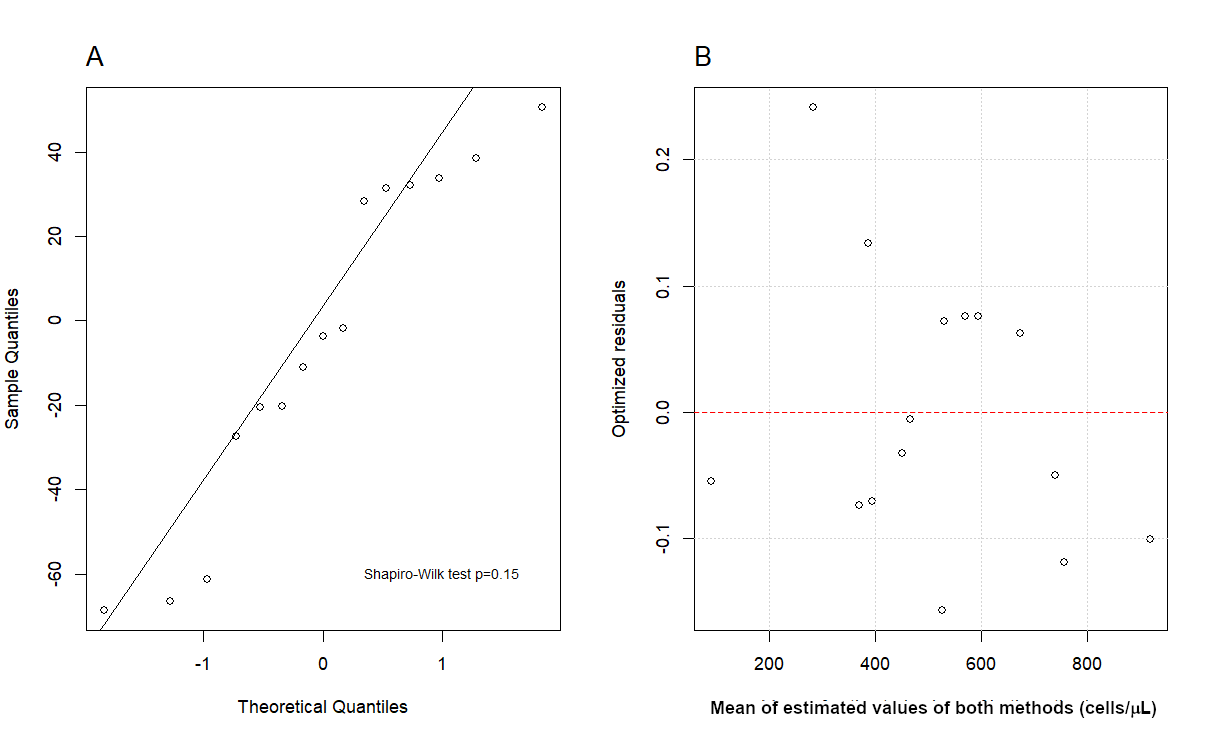

Supplement: S2 Fig — (A) Normality of residuals. (B) Homogeneity of variance. (TIF) [file pone.0345611.s002.tif]

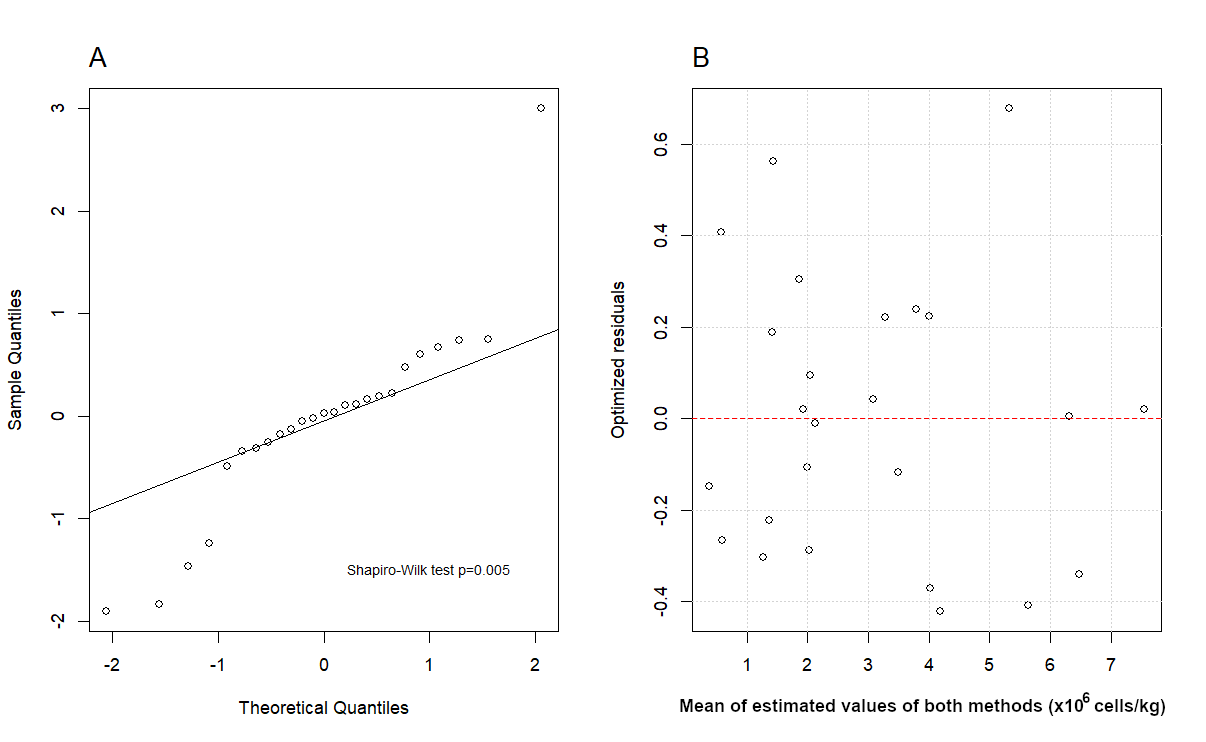

Supplement: S3 Fig — (A) Normality of residuals. (B) Homogeneity of variance. (TIF) [file pone.0345611.s003.tif]

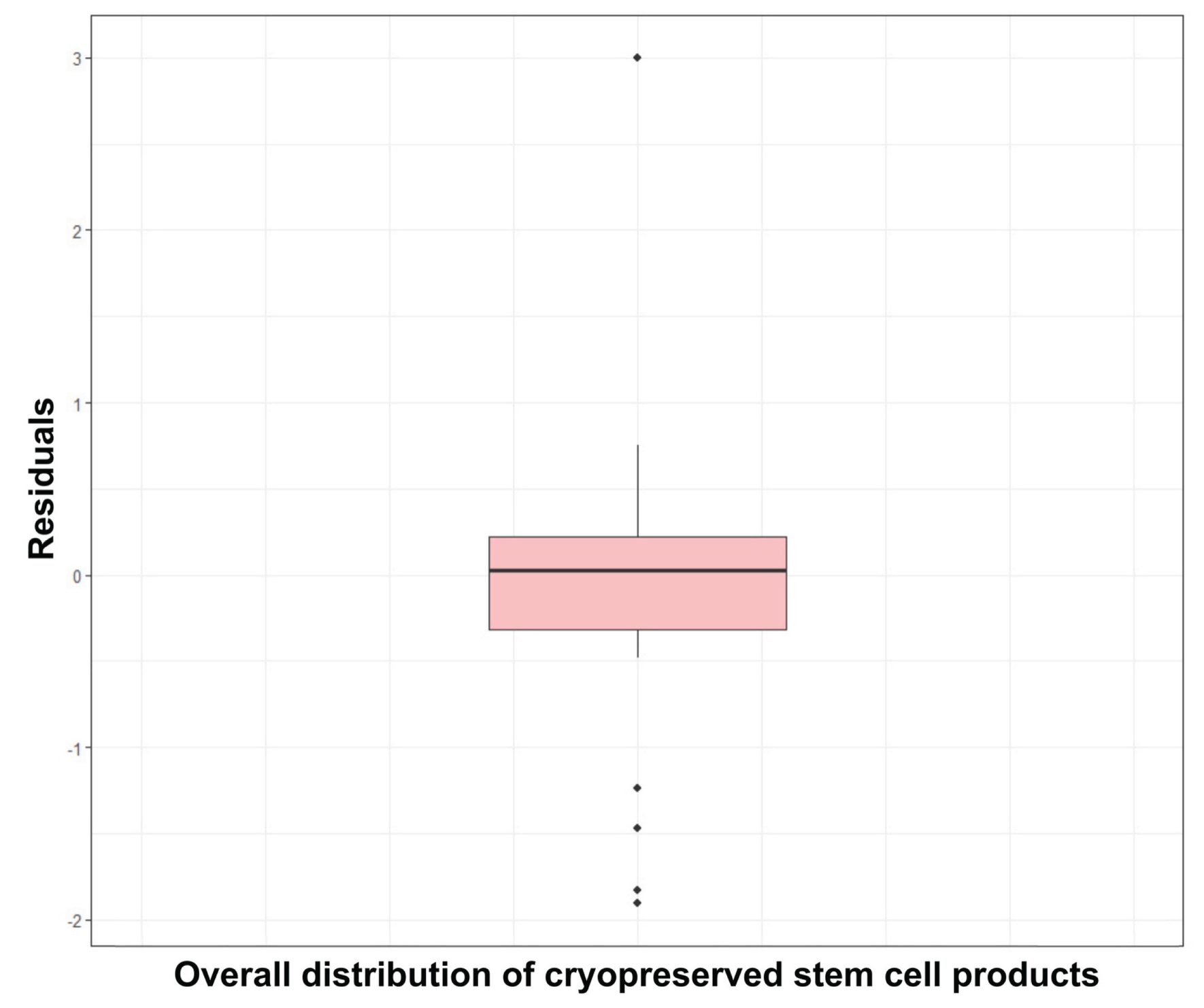

Supplement: S4 Fig — (TIF) [file pone.0345611.s004.tif]

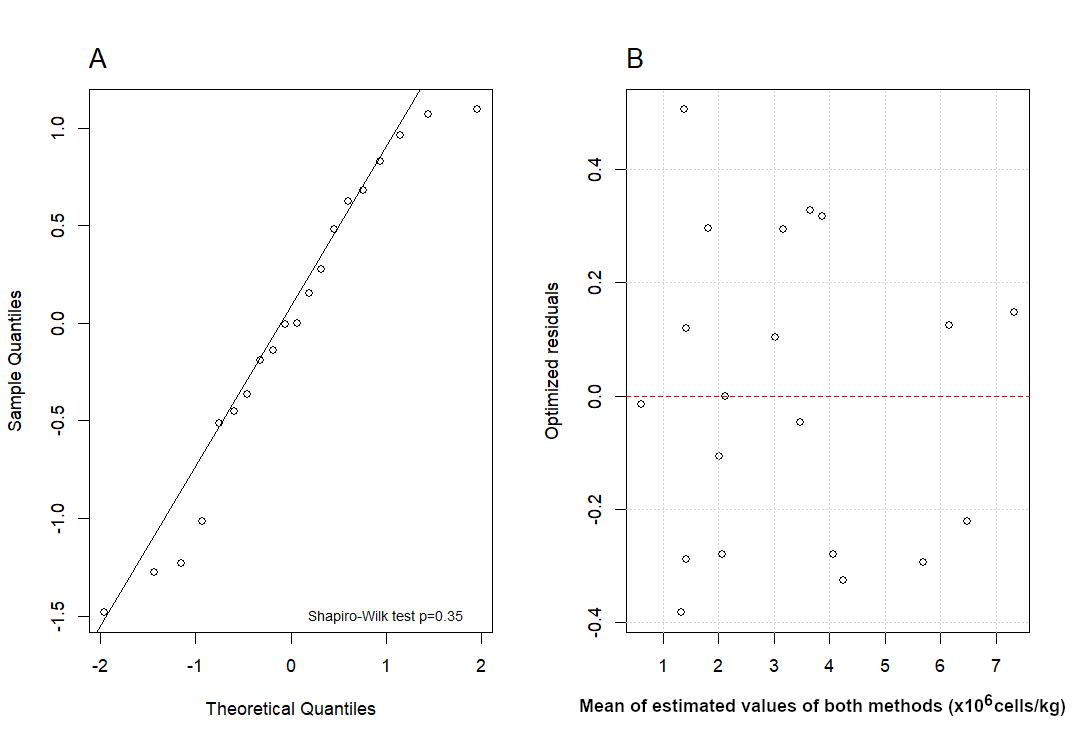

Supplement: S5 Fig — (A) Normality of residuals. (B) Homogeneity of variance. (TIF) [file pone.0345611.s005.tif]
